# Tunneling Quantum Dynamics in Ammonia

**DOI:** 10.3390/ijms22158282

**Published:** 2021-07-31

**Authors:** Ciann-Dong Yang, Shiang-Yi Han

**Affiliations:** 1 Department of Aeronautics and Astronautics, National Cheng Kung University, Tainan 701, Taiwan; 2 Department of Applied Physics, National University of Kaohsiung, Kaohsiung 811, Taiwan; syhan.taiwan@gmail.com

**Keywords:** ammonia, quantum molecular dynamics, quantum Hamilton mechanics, tunneling dynamics, quantum trajectory

## Abstract

Ammonia is a well-known example of a two-state system and must be described in quantum-mechanical terms. In this article, we will explain the tunneling phenomenon that occurs in ammonia molecules from the perspective of trajectory-based quantum dynamics, rather than the usual quantum probability perspective. The tunneling of the nitrogen atom through the potential barrier in ammonia is not merely a probability problem; there are underlying reasons and mechanisms explaining why and how the tunneling in ammonia can happen. Under the framework of quantum Hamilton mechanics, the tunneling motion of the nitrogen atom in ammonia can be described deterministically in terms of the quantum trajectories of the nitrogen atom and the quantum forces applied. The vibrations of the nitrogen atom about its two equilibrium positions are analyzed in terms of its quantum trajectories, which are solved from the Hamilton equations of motion. The vibration periods are then computed by the quantum trajectories and compared with the experimental measurements.

## 1. Introduction

Tunneling, one of the most fascinating and mysterious phenomenon in the microscopic world, has benefitted our daily life for decades. Even though quantum mechanics has provided some useful information about tunneling, we still barely know how it works. As new technology equips quantum devices with more potential advanced usages and applications [1,2,3,4], the current knowledge has reached its limitation. More studies have begun to extend this limitation to the edge of the microscopic world [5,6,7,8]. To seek the underlying physics and mechanism of tunneling, various approaches have been studied [9,10,11,12,13,14,15,16,17,18,19,20]. One of these approaches is the trajectory interpretation of quantum mechanics, which regards the wave function as an ensemble of trajectories [21,22,23,24,25,26,27,28].

The trajectory interpretation of quantum mechanics provides an ontological perspective to view the microscopic world. By means of an ensemble of trajectories, particle properties and wave properties in quantum theory can be connected [29,30]. In recent years, the discussion of the quantum trajectory has been extended to the complex space [31,32,33,34,35,36]. Higher dimensions provide leverage in tackling unsolved quantum issues and explaining more quantum phenomena [37,38,39,40,41,42,43,44,45,46]. Underlying the framework of complex trajectory interpretation, tunneling dynamics have been provided and studied. Levkov [47] closely inspected tunneling trajectory in a chaotic model in the complex domain. Yang [48] presented tunneling dynamics in the complex space, revealing a smooth trajectory which continuously connects the classical trajectory and tunneling trajectory. John [49] evaluated the reflection probability in terms of the reflected and incident complex trajectories.

A series of experiments verify the reality of quantum trajectories, and some even show the importance and necessity of the consideration of the complex domain in the quantum system [50,51,52,53,54,55,56]. Following the proposal of a weak value [57], the measurement and observation of the quantum system can be carried out to reveal the reality of the quantum realm. Under the minimum degree of interference in the quantum system, this weak measurement endows the complex eigenvalues with the physical meanings. Various researches have extensively studied the fundamental mechanisms of the weak value, pointing out that the imaginary part of the weak value is significantly important to quantum observations [58,59,60,61,62]. In the latest research, this complex number is reported as an essential element in inspecting quantum systems [63,64]. With support from both theoretical and experimental evidences, the complex trajectory interpretation is gradually becoming one of the most conceivable interpretations of quantum mechanics. In the present research, we will apply the complex trajectory interpretation to a practical quantum system involving ammonia and its inversion state in order to analyze the tunneling dynamics between them.

Ammonia is formed in the shape of a pyramid, with three hydrogen atoms situated on an equilateral triangle plane with the nitrogen atom on the apex. The pyramid inverts as the nitrogen atom changes position from one equilibrium point to another via the tunneling effect. This inversion flip-flops repeatedly with the tunneling rate $2.4\times 10^{12}$ Hz, which is calculated by solving the Schrödinger equation in the double-well potential with the WKB method [65], having an experimental value of $2.3786\times 10^{10}$ Hz.

From the viewpoint of quantum mechanics, it is probably that at a given time the nitrogen atom is situated on either side of the equilateral triangle plane formed by the three hydrogen atoms. In other words, it is partly on both sides at the same time in a quantum mechanical sense. However, the frequency of the nitrogen atom flips back and forth has been experimentally observed [66]. Does this imply that the nitrogen atom is wholly on one side or on the other at any given instant? In this paper, we will analyze the tunneling dynamics in ammonia by means of the complex trajectory interpretation of quantum mechanics. It seems that the tunneling trajectory might provide some convincing answers to the above question.

This paper is organized as follows. Section 2 introduces the complex trajectory interpretation of quantum mechanics in terms of quantum Hamilton mechanics. In order to analyze the tunneling trajectory in the ammonia molecule, we first obtain its vibrational wave functions in Section 3. We then use quantum Hamilton mechanics together with the obtained vibrational wave functions in Section 4 and Section 5 to discuss the tunneling dynamics of the nitrogen atom under the action of the single-well potential and the double-well potential in the ammonia molecule. Section 6 presents the tunneling dynamics of the nitrogen atom vibrating between the ammonia state and the ammonia inversion state, and the computed tunneling frequency and tunneling range are compared with the measurement data in good agreement.

## 2. Quantum Hamilton Mechanics

We begin this section with a brief introduction to quantum Hamilton mechanics [32]. Consider a quantum particle in the 3-dimensional complex space, whose position is described by the complex coordinate $q=\left(q_{1},q_{2},q_{3}\right)$ with $q=q_{R}+iq_{I}\in {\mathbb{C}}^{3}$. The Schrödinger equation with the complex coordinate q

(1) $$i\hbar \frac{\partial \Psi \left(t,q\right)}{\partial t}=-\frac{\hbar^{2}}{2m}\nabla^{2}\Psi \left(t,q\right)+V\Psi \left(t,q\right)$$

can be recast into the quantum Hamilton–Jacobi equation

(2) $$\frac{\partial S\left(t,q\right)}{\partial t}+{\left[\frac{1}{2m}p\cdot p+V\left(t,q\right)+\frac{1}{2mi}\nabla \cdot p\right]}_{p=\nabla S}=\frac{\partial S\left(t,q\right)}{\partial t}+{\left.H\left(t,q,p\right)\right|}_{p=\nabla S}=0$$

via the relation

(3) $$\Psi \left(t,q\right)=e^{iS\left(t,q\right)/\hbar }$$

where $S\left(t,q\right)$ is the action function and $\Psi \left(t,q\right)$ is the wave function. The quantum Hamiltonian $H\left(t,q,p\right)$ is the sum of the particle’s kinetic energy $p^{2}/2m$, the external potential $V\left(t,q\right)$, and the quantum potential $Q\left(t,q\right)$.

(4) $$H\left(t,q,p\right)=\frac{1}{2m}p\cdot p+V\left(t,q\right)+Q\left(t,q\right)$$

The quantum potential Q in (4) is defined by the following expressions:

(5) $$Q\left(t,q\right)={\left.\frac{\hbar }{2mi}\nabla \cdot p\right|}_{p=\nabla S}=\frac{\hbar }{2mi}\nabla^{2}S\left(t,q\right)=-\frac{\hbar^{2}}{2m}\nabla^{2}\ln\Psi \left(t,q\right)$$

The quantum potential is state-dependent and can be determined immediately if the wave function $\Psi \left(t,q\right)$ is known. Without the quantum potential $Q\left(t,q\right)$, the Hamiltonian (4) reduces to the classical Hamiltonian. Hence, it is safe to say that quantum potential plays the key role in the quantum world, rendering everything different from our familiar classical world.

The Hamilton equations of motion in the complex space are derived from the quantum Hamiltonian (4) as:

(6) $${\dot{q}}_{j}=\frac{\partial H}{\partial p_{j}}=\frac{p_{j}}{m},\;\;\;\;q_{j}\left(t_{0}\right)=q_{j}^{0}\in \mathbb{C},\;\;\;j=1,\;2,\;3$$

(7) $${\dot{p}}_{j}=-\frac{\partial H}{\partial q_{j}}=-\frac{\partial }{\partial q_{j}}\left(V+Q\right),\;\;\;\;p_{j}\left(t_{0}\right)=p_{j}^{0}\in \mathbb{C},\;\;\;j=1,\;2,\;3$$

The particle’s momentum p=∇S can be expressed in terms of the wave function through the relation (3):

(8) $$p_{j}=\frac{\partial S\left(t,q\right)}{\partial q_{j}}=\frac{\hbar }{i}\frac{1}{\Psi \left(t,q\right)}\frac{\partial \Psi \left(t,q\right)}{\partial q_{j}}$$

With (8), the particle’s equations of motion (6) can be represented as a function of $\Psi \left(t,q\right)$,

(9) $${\dot{q}}_{j}=\frac{p_{j}}{m}=\frac{\hbar }{im}\frac{1}{\Psi \left(t,q\right)}\frac{\partial \Psi \left(t,q\right)}{\partial q_{j}},\;\;\;j=1,\;2,\;3$$

By solving (9), the particle’s trajectory in the complex space can then be obtained. In the formalism of quantum Hamilton mechanics, the wave function $\Psi \left(t,q\right)$ with $\;q\in {\mathbb{C}}^{3}$ can provide detailed dynamic information of the particle in addition to the probability density ${\left|\Psi \left(t,q\right)\right|}^{2}$.

The combination of (8) and (9) gives the quantum Newton’s law in the complex space,

(10) $$m{\ddot{q}}_{j}=-\frac{\partial }{\partial q_{j}}\left(V+Q\right)=-\frac{\partial }{\partial q_{j}}V_{\mathrm{Total}}$$

where $V_{\mathrm{Total}}$ is the total potential containing the external potential V and the quantum potential Q:

(11) $$V_{\mathrm{Total}}=V+Q=V\left(t,q\right)-\frac{\hbar^{2}}{2m}\nabla^{2}\ln\Psi \left(t,q\right)$$

If $\Psi \left(t,q\right)$ is an eigenstate with eigen energy, E, it can be expressed by $\Psi \left(t,q\right)=\psi \left(q\right)e^{-i\left(E/\hbar \right)t}$ and the accompanying action function becomes $S\left(t,q\right)=-i\hbar \ln\psi \left(q\right)-Et$. Substituting $S\left(t,q\right)$ into the quantum Hamilton–Jacobi Equation (2) yields $H\left(t,q,p\right)=p^{2}/2m+V+Q=-\partial S/\partial t=E=\mathrm{constant}$, and

(12) $$V_{\mathrm{Total}}=V+Q=E-\frac{p^{2}}{2m}=E+\frac{\hbar^{2}}{2m}{\left(\nabla \ln\psi \right)}^{2}$$

The use of the above $V_{\mathrm{Total}}$ in (10) leads to the quantum Newton’s law

(13) $$m{\ddot{q}}_{j}=-\frac{\partial V_{\mathrm{Total}}}{\partial q_{j}}=-\frac{\hbar^{2}}{2m}\frac{\partial }{\partial q_{j}}{\left(\nabla \ln\psi \right)}^{2},\;\;q_{j}\in \mathbb{C},\;\;j=1,\;2,\;3$$

In quantum mechanics, the squared magnitude of $\psi \left(q\right)$ determines the probability of finding a particle in specific regions. In quantum Hamilton mechanics, the wave function $\psi \left(q\right)$ provides the total potential $V_{\mathrm{Total}}$ to yield the equation of motion (13). The interrelationship of the total potential $V_{\mathrm{Total}}$ and probability density function ${\left|\psi \right|}^{2}$ can be established from (12) as follows:

(14) $$\left|V_{\mathrm{Total}}-E\right|=\left|\frac{\hbar^{2}}{2m}{\left(\nabla \ln\psi \right)}^{2}\right|=\frac{\hbar^{2}}{2m}\frac{{\left|\nabla \psi \right|}^{2}}{{\left|\psi \right|}^{2}}\;$$

which shows that the height of the total potential, $V_{\mathrm{Total}}-E$, is inversely proportional to ${\left|\psi \right|}^{2}$. This reflects the very truth that a greater probability of finding a particle corresponds to a lower total potential. In Section 4 and Section 5, we will inspect this interrelationship closely by demonstrating the quantum dynamics of ammonia in different eigenstates.

## 3. Vibrational Eigenfunctions of Ammonia

**Figure 1:**
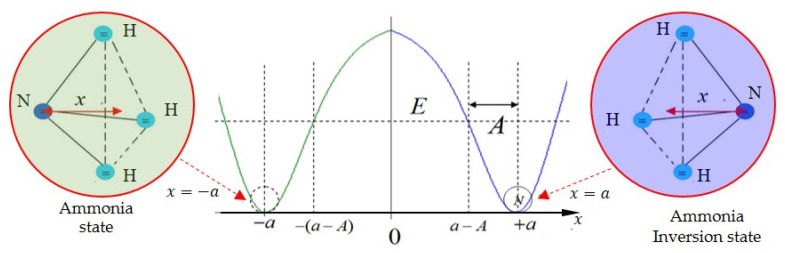
The two equilibrium states of the ammonia molecule and their related positions in the double-well potential. The ammonia molecule has the shape of a pyramid, where the three hydrogen atoms form the equilateral triangle and the nitrogen atom is positioned at the apex. The distance of the nitrogen atom to the equilateral triangle plane is denoted by x, and the double-well potential is symmetric to x=0. The positions x=±a are the two equilibrium points of the potential, recognized as the ammonia state and the ammonia inversion state, respectively. The positions $x=\pm \left(a-A\right)$ are the classical turning points, where the nitrogen atom’s kinetic energy is equal to the potential barrier.

The nitrogen atom in ammonia vibrates in a double-well potential, which can be expressed by the following function [67]:

(15) $$V\left(x\right)=\left\{\begin{array}{c} B\tan h\left(x/d-k\right)-C{\sec h}^{2}\left(x/d-k\right)=V^{+}\left(x\right),\;\;\;\;\;x\geq 0\\ -B\tan h\left(x/d+k\right)-C{\sec h}^{2}\left(x/d+k\right)=V^{-}\left(x\right),\;\;x\leq 0 \end{array}\right.$$

where B, C, d, and k are parameters determined by the experimental data. The coordinate x is the distance of the nitrogen atom to the plane formed by the three hydrogen atoms, as displayed in Figure 1. The double-well potential is composed of two single-well potentials. In this study, the Rosen–Morse potential is applied to the single-well potential model,

(16) $$V_{s}\left(x\right)=B\tan h\left(x/d\right)-C{\sec h}^{2}\left(x/d\right)$$

By shifting the above single-well potential with a distance k to the left-hand side and right-hand side of the x=0 axis, we obtain the double-well potentials, $V^{+}\left(x\right)=V_{s}\left(x/d-k\right)$ and $V^{-}\left(x\right)=V_{s}\left(-x/d-k\right)$, as expressed by (15). This double-well potential is symmetric to the x=0 axis and is continuous at x=0 by noting $V\left(0^{-}\right)=V\left(0^{+}\right)=-B\tan h\left(k\right)-C{\sec h}^{2}\left(k\right)$. An additional condition, $\tan h\left(k\right)=B/2C$, should be satisfied as having the continuity of the first derivative of $V\left(x\right)$ at x=0.

The Schrödinger equation describing the motion of the nitrogen atom in the double-well potential $V\left(x\right)$ reads

(17) $$-\frac{\hbar^{2}}{2M}\frac{d^{2}\Psi \left(x\right)}{dx^{2}}+\left(V\left(x\right)-W\right)\Psi \left(x\right)=0$$

whose solution $\Psi \left(x\right)$ can be approximately given by the linear combination of the wave function $\psi \left(x\right)$, which is the solution of the Schrödinger equation with the single-well potential $V_{s}\left(x\right)$:

(18) $$-\frac{\hbar^{2}}{2M}\frac{d^{2}\psi \left(x\right)}{dx^{2}}+\left(V_{s}\left(x\right)-E\right)\psi \left(x\right)=0$$

The mass M in (17) and (18) is the effective mass of the nitrogen atom N in the ammonia molecule $NH_{3}$, defined as $M=3m_{H}m_{N}/\left(3m_{H}+m_{N}\right)$ where $m_{H}$ and $m_{N}$ denote the mass of the hydrogen atom and nitrogen atom, respectively. By defining the following dimensionless variables:

(19) $$z=x/d,\;\;\varepsilon =-E/g,\;\;\beta =B/g,\;\;\gamma =C/g,\;\;g=\hbar^{2}/2Md^{2}$$

one can express the Schrödinger Equation (18) in the dimensionless form:

(20) $$\frac{d^{2}\psi \left(z\right)}{dz^{2}}+\left(-\varepsilon -\beta \tan hz+\gamma {\sec h}^{2}z\right)\psi \left(z\right)=0$$

The solution to (20) can be expressed analytically as [67]

(21) $$\psi \left(z\right)=e^{az}{\left(\cosh z\right)}^{-b}F\left(z\right)$$

where

(22) $$F\left(u\right)=\mathrm{hypergeom}\left(\left[b+1/2-\sqrt{\gamma +1/4},b+1/2+\sqrt{\gamma +1/4}\right],\left[a+b+1\right],u\right)$$

and

(23) $$u=\frac{1}{2}\left(1+\tan hz\right),\;\;a=-\frac{1}{2}\left(\sqrt{\varepsilon +\beta }-\sqrt{\varepsilon -\beta }\right),\;\;b=\frac{1}{2}\left(\sqrt{\varepsilon +\beta }-\sqrt{\varepsilon -\beta }\right)$$

The hypergeometric function $F\left(u\right)$ approaches infinity when u approaches unity, unless

(24) $$b+1/2-\sqrt{\gamma +1/4}=-n,\;\;\;\;\;\;\;n=0,\;1,\;2,\cdots$$

(24) gives the boundedness condition for $F\left(u\right)$ and quantizes the constant a and b as

(25) $$b_{n}=\sqrt{\gamma +1/4}-n-1/2,\;\;\;\;\;\;\;n=0,1,2,\cdots$$

(26) $$a_{n}=-\beta /2b_{n}=-\beta /\left(\sqrt{4\gamma +1}-2n-1\right),\;\;\;\;n=0,1,2,\cdots$$

and the corresponding eigenfunction ψ_n is given by (21). The quantization of a and b result in the energy quantization:

(27) $$-E_{n}=g\varepsilon_{n}=g\left(a_{n}^{2}+b_{n}^{2}\right)=g\left[\frac{1}{4}{\left(\sqrt{4\gamma +1}-2n-1\right)}^{2}+\beta^{2}{\left(\sqrt{4\gamma +1}-2n-1\right)}^{-2}\right]$$

The energy has an upper bound, beyond which the ammonia molecule will be dissociated. We can find the allowable range of n according to the condition $a_{n}+b_{n}=\sqrt{\varepsilon_{n}-\beta }\geq 0$, which leads to

(28) $$n\leq \sqrt{\gamma +1/4}-\sqrt{\beta /2}-1/2$$

For each single-well solution $\psi_{n}$ to (18), there are two double-well solutions $\Psi_{n}^{+}\left(z\right)$ and $\Psi_{n}^{-}\left(z\right)$ to (17), as given by

(29) $$\Psi_{n}^{+}\left(z\right)=\frac{1}{\sqrt{2}}\left[\psi_{n}\left(z-k\right)+\psi_{n}\left(-z-k\right)\right]$$

(30) $$\Psi_{n}^{-}\left(z\right)=\frac{1}{\sqrt{2}}\left[\psi_{n}\left(z-k\right)+\psi_{n}\left(-z-k\right)\right]$$

**Figure 2:**
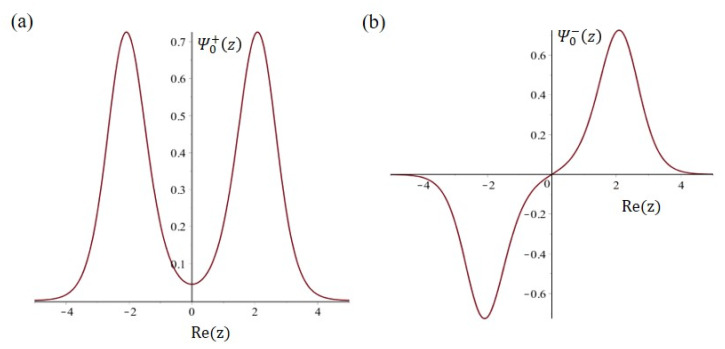
The two ground-state wave functions of the nitrogen atom in the double-well potential. (**a**) $\Psi_{0}^{+}\left(z\right)=\Psi_{\mathrm{even}}\left(z\right)$ has even symmetry. (**b**) $\Psi_{0}^{-}\left(z\right)=\Psi_{\mathrm{odd}}\left(z\right)$ has odd symmetry.

It can be seen from Figure 2 that $\Psi_{n}^{+}\left(z\right)$ has even symmetry and $\Psi_{n}^{-}\left(z\right)$ has odd symmetry. The associated energy levels of $\Psi_{n}^{+}\left(z\right)$ and $\Psi_{n}^{-}\left(z\right)$ are $W_{n}^{\pm }$, which in dimensionless form are given by

(31) $$\frac{W_{n}^{\pm }}{g}=\varepsilon_{n}+\int_{0}^{\infty }\psi_{n}^{2}\left(z+k\right)V\left(z\right)dz\pm \int_{0}^{\infty }\psi_{n}\left(z-k\right)V\left(z\right)\psi_{n}\left(-z-k\right)dz\;\;\;\;\;$$

Under the framework of quantum Hamilton mechanics, we see exactly how the nitrogen atom moves in the double-well potential, and how it transits from one side to the other side via its trajectory solved from (9) with wave functions given by (29) and (30). To have the computation close to the actual situation as much as possible, all parameters are obtained from the experimental data, including the energy gap between the ground state and the first excited state, $E_{1}-E_{0}=950{\;\mathrm{cm}}^{-1}$ and the inter-pair separation in the ground state and the first excited state, $∆W_{0}=W_{0}^{+}-W_{0}^{-}=0.8118\;{\mathrm{cm}}^{-1}$, $∆W_{1}=W_{1}^{+}-W_{1}^{-}=33\;{\mathrm{cm}}^{-1}$, where ${\mathrm{cm}}^{-1}$ is an energy unit defined by $1{\;\mathrm{cm}}^{-1}=hc\;\mathrm{joule}=1.988\times 10^{-23}\;\mathrm{joule}$. The parameters B, C, d, and k can be determined from the above three experimental data via the following three relations:

(32) $$\frac{E_{1}-E_{0}}{g}=2\sqrt{\gamma +1/4}-2-\frac{\beta^{2}}{4}\frac{\sqrt{4\gamma +1}-2}{{\left(\gamma +1-\sqrt{4\gamma +1}\right)}^{2}}$$

(33) $$\frac{∆W_{0}}{g}=\frac{W_{0}^{+}-W_{0}^{-}}{g}=2\int_{0}^{\infty }\psi_{0}\left(z-k\right)\;V\left(z\right)\psi_{0}\left(-z-k\right)dz$$

(34) $$\frac{∆W_{1}}{g}=\frac{W_{1}^{+}-W_{1}^{-}}{g}=2\int_{0}^{\infty }\psi_{1}\left(z-k\right)\;V\left(z\right)\psi_{1}\left(-z-k\right)dz$$

where $\psi_{0}$ and $\psi_{1}$ are wave functions of the ground state and the first excited state given by (21):

(35) $$\psi_{0}\left(z\right)=e^{a_{0}z}{\left(\cosh z\right)}^{-b_{0}}$$

(36) $$\psi_{1}\left(z\right)=e^{a_{1}z}{\left(\cosh z\right)}^{-b_{1}}\left[1-\frac{\sqrt{4\gamma +1}-1}{a_{1}+b_{1}+1}\left(1+\tan hz\right)/2\right]$$

Due to the limited available data, the three relations (32)–(34) are not enough to determine the four parameters B, C, d, and k uniquely, but only their ranges can be identified: $0\leq B\leq 1000\;{\mathrm{cm}}^{-1}$, $2200{\;\mathrm{cm}}^{-1}\leq C\leq 3000\;{\mathrm{cm}}^{-1}$, 16 pm≤d≤18.5 pm, and 2.20≤k≤2.24. Numerical values picked up from the above ranges will be employed in the following calculation of quantum trajectories.

## 4. Nitrogen Dynamics in Single-Well Potential

The wave function stores a lot of information of a quantum state, including information that cannot be provided by the quantum probability, such as the tunneling trajectory, tunneling time, quantum potential, and so on. In this section we will consider nitrogen dynamics in the single-well potential in order to find its trajectory in the complex plane. The ground-state wave function $\psi_{0}\left(z\right)=e^{a_{0}z}{\left(\cosh z\right)}^{-b_{0}}$ for the single-well potential yields a pair of the ground-state wave functions for the double-well potential according to (29) and (30):

(37) $$\Psi_{0}^{+}\left(z\right)=\frac{1}{\sqrt{2}}\left[e^{a_{0}\left(z-k\right)}{\left(\cosh\left(z-k\right)\right)}^{-b_{0}}+e^{a_{0}\left(-z-k\right)}{\left(\cosh(z+k)\right)}^{-b_{0}}\right]$$

(38) $$\Psi_{0}^{-}\left(z\right)=\frac{1}{\sqrt{2}}\left[e^{a_{0}\left(z-k\right)}{\left(\cosh\left(z-k\right)\right)}^{-b_{0}}-e^{a_{0}\left(-z-k\right)}{\left(\cosh(z+k)\right)}^{-b_{0}}\right]$$

For the purposes of simplification, all functions are considered in dimensionless forms.

The ground-state equation of motion for the nitrogen atom in the single-well potential is given by (9):

(39) $$\frac{dz}{d\tau }=\frac{1}{i}\frac{d\ln\psi_{0}\left(z\right)}{dz}=-i\left(a_{0}-b_{0}\tan hz\right),\;\;\;\;\;\;\;\tau =\frac{\hbar }{Md^{2}}t$$

which has an equilibrium point $z_{\mathrm{eq}}={\tan h}^{-1}\left(a_{0}/b_{0}\right)$ by letting dz/dτ=0. Let us express the equilibrium point in terms of variables β and γ with the help of (25) and (26):

(40) $$z_{\mathrm{eq}}=-{\tan h}^{-1}\left[\frac{2\beta }{{\left(\sqrt{4\gamma +1}-1\right)}^{2}}\right].\;\;\;\;$$

**Figure 3:**
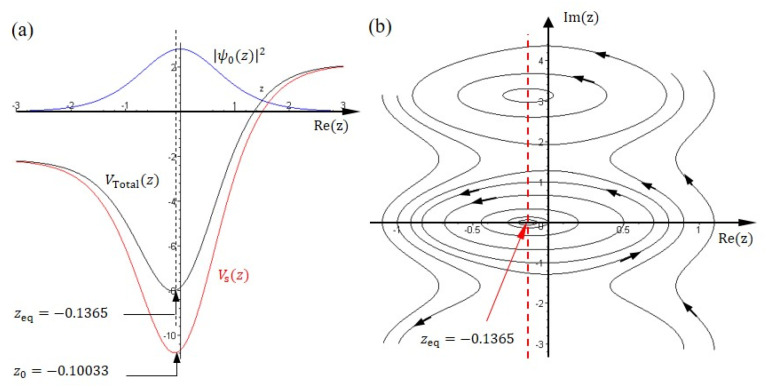
(**a**) The plots of ${\left|\psi_{0}\left(z\right)\right|}^{2}$, $V_{s}\left(z\right)$ and $V_{\mathrm{Total}}\left(z\right)$ for the single-well potential. The parameters are chosen as follows: $B=500{\;\mathrm{cm}}^{-1}$, $C=2500{\;\mathrm{cm}}^{-1}$, d=17 pm, and k=2.22. The maximum point of ${\left|\psi_{0}\left(z\right)\right|}^{2}$ at Rez=−0.1365 coincides with the minimum point of the potential $V_{\mathrm{Total}}\left(z\right)$, but is different from the minimum point of $V_{s}\left(z\right)$ at Rez= −0.10033. (**b**) The complex trajectories of the nitrogen atom solved from (39) show that the equilibrium point $z_{eq}$ is located at the minimum point of the potential $V_{\mathrm{Total}}\left(z\right)$, which is also the point with the maximum probability.

The single-well potential, $V_{s}\left(z\right)=\beta \tan h\left(z\right)-\gamma {\sec h}^{2}\left(z\right)$, has a minimum located at $z_{0}=-{\tan h}^{-1}\left(\beta /2\gamma \right)$ with the minimum value $V_{s}\left(z_{0}\right)=-\beta^{2}+4\gamma^{2}/4\gamma$. It is clear to see that there is a deviation between the equilibrium point $z_{\mathrm{eq}}$ and the minimum point $z_{0}$, as Figure 3a shows. The quantum potential is the origin of this deviation since the equilibrium point $z_{\mathrm{eq}}$ represents the minimum point of the total potential $V_{\mathrm{Total}}\left(z\right)=V_{s}\left(z\right)+Q\left(z\right)$. From (5) we have the quantum potential for the ground state,

(41) $$Q\left(z\right)=-\frac{d^{2}\ln\psi_{0}\left(z\right)}{dz^{2}}=-b_{0}{\sec h}^{2}z$$

and the complete expression of the total potential for the ground state then reads,

(42) $$V_{\mathrm{Total}}\left(z\right)=\beta \tan hz+\left(b_{0}-\gamma \right){\;\sec h}^{2}z$$

The minimum of the total potential can be found by letting $dV_{\mathrm{Total}}\left(z\right)/dz=0$:

(43) $$\beta {\sec h}^{2}z\left(1-b_{0}\tan hz/a_{0}\right)=0$$

from which the minimum point $z_{\min}$ is found to be identical to the equilibrium point $z_{\mathrm{eq}}$ as shown in (40). One notes that if γ≫1, then (40) gives $z_{\mathrm{eq}}=-{\tan h}^{-1}\left(\beta /2\gamma \right)$ that equals the minimum point $z_{0}$ of the potential $V_{s}\left(z\right)$. 

As shown in Figure 3, the maximum probability density ${\left|\psi_{0}\left(z\right)\right|}_{\max}^{2}$ locates at the position $d\psi_{0}/dz=0$, which is just the equilibrium position $z_{\mathrm{eq}}$ and the minimum point $z_{\min}$ of the total potential $V_{\mathrm{Total}}\left(z\right)$, but not the minimum point $z_{0}$ of the potential $V_{s}\left(z\right)$. Probability is the main character throughout quantum mechanics and is the main measurement outcome of the quantum world. Here, we can see that the role of the probability can be replaced by the quantum potential, which is one of the compositions of the quantum Hamiltonian as appeared in (4). Accordingly, the quantum world may not be purely probabilistic, but rather classical with causal essence hidden within quantum mechanics. We are able to discover some causal characteristics of the quantum world by manifesting various classical counterpart features underlying the formulation of quantum Hamilton mechanics.

Let us calculate the vibration period of the Nitrogen atom in ammonia in a classical manner. By using the Newton’s second law (10) in a complex domain, the force acting on the nitrogen atom by the total potential is

(44) $$\frac{d^{2}z}{d\tau^{2}}=-\frac{dV_{\mathrm{Total}}\left(z\right)}{dz}$$

We can further obtain the force constant evaluated at $z_{\min}$:

(45) $$\bar{K}={\left.\frac{d^{2}V_{\mathrm{Total}}\left(z\right)}{dz^{2}}\right|}_{z=z_{\min}}=\frac{2}{b_{0}^{2}}{\left(b_{0}^{2}-a_{0}^{2}\right)}^{2},\;\;\;\;\;\;\;\;\;K=\frac{\hbar^{2}}{2Md^{2}}\bar{K}$$

where we have used the relation $a_{0}b_{0}=-\beta /2,$ and the bar denotes the dimensionless symbol. According to the classical formula of a vibration period, $T=2\pi \sqrt{M/K}$, we have the dimensionless vibration period $\bar{T}$,

(46) $$\bar{T}=\frac{\hbar }{Md^{4}}T=\frac{2\pi b_{0}}{b_{0}^{2}-a_{0}^{2}}$$

In the macroscopic world we can visually observe any kinds of vibration propagating with media. In the formulation of quantum Hamilton mechanics, we can visualize the vibration pattern too in terms of the nitrogen atom’s trajectories in a complex plane. Figure 3b illustrates some trajectories solved from (39) in the complex z−plane with different initial positions. The parameters are chosen as: $B=500{\;\mathrm{cm}}^{-1}$, $C=2500{\;\mathrm{cm}}^{-1}$, d=17 pm*,* and k=2.22. One initial position $z\left(0\right)$ corresponds to one trajectory in the complex z−plane. Two types of trajectory can be found in Figure 3b: closed trajectories and open trajectories. When the initial position is close to the equilibrium point, the nitrogen atom will oscillate around the center, $z_{\mathrm{eq}}=-0.1365$, in the lower part of the potential well and forms a closed trajectory. The total energy of the nitrogen atom is given by the quantum Hamiltonian (4):

(47) $$H=E_{k}\left(z\right)+V_{s}\left(z\right)+Q\left(z\right)\;=-{\left(a_{0}-b_{0}\tan hz\right)}^{2}-2a_{0}b_{0}\tan hz-b_{0}^{2}{\mathrm{sech}}^{2}z\;=-\left(a_{0}^{2}+b_{0}^{2}\right)=-\varepsilon_{0}=\mathrm{constant}.\;$$

This energy value can be confirmed by inserting n=0 into (27). From (47), we can see that the total energy cannot be a constant if the quantum potential is not considered in the Hamiltonian.

## 5. Tunneling Dynamic in Stationary States

### 5.1. Tunneling Trajectory in the Ground State

The double-well potential (15) with the same parameters assigned in Section 4, i.e., $B=500{\;\mathrm{cm}}^{-1}$, $C=2500{\;\mathrm{cm}}^{-1}$, d=17 pm*,* and k=2.22, can be expressed by

(48) $$V\left(z\right)=\left\{\begin{array}{c} 2.1332\tan h\left(z-k\right)-10.666{\sec h}^{2}\left(z-k\right)=V^{+}\left(z\right),\;\;\;\;\;z\geq 0\\ -2.1332\tan h\left(z+k\right)-10.666{\sec h}^{2}\left(z+k\right)=V^{-}\left(z\right),\;\;z\leq 0 \end{array}.\right.$$

(49) $$\Psi_{0}^{+}\left(z\right)=\frac{1}{\sqrt{2}}\left(e^{-0.3804\left(z-k\right)}{\left(\cosh\left(z-k\right)\right)}^{-2.8039}+e^{-0.3804\left(-z-k\right)}{\left(\cosh(z+k)\right)}^{-2.8039}\right)$$

(50) $$\Psi_{0}^{-}\left(z\right)=\frac{1}{\sqrt{2}}\left(e^{-0.3804\left(z-k\right)}{\left(\cosh\left(z-k\right)\right)}^{-2.8039}-e^{-0.3804\left(-z-k\right)}{\left(\cosh(z+k)\right)}^{-2.8039}\right)$$

(37) and (38) is a pair of ground-state wave functions.

**Figure 4:**
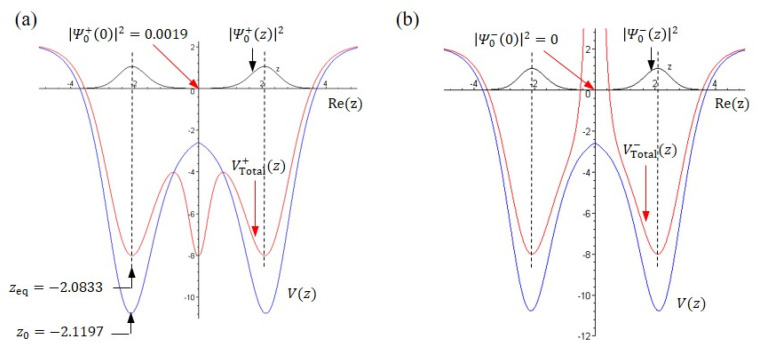
The spatial distributions of the probability density ${\left|\Psi_{0}^{\pm }\left(z\right)\right|}^{2}$, the double-well potential $V\left(z\right)$, and the total potential $V_{\mathrm{Total}}^{\pm }\left(z\right)$ in the ground state. (**a**) The maximum point of the probability density ${\left|\Psi_{0}^{+}\left(z\right)\right|}^{2}$ at Rez=±2.08331 coincides with the minimum point of the total potential $V_{\mathrm{Total}}^{+}\left(z\right)$, but is different from the minimum point of the double-well potential $V\left(z\right)$ at Rez=±2.11966. The probability ${\left|\Psi_{0}^{+}\left(z\right)\right|}^{2}$ at the origin is ${\left|\Psi_{0}^{+}\left(0\right)\right|}^{2}=0.001938\neq 0$ which corresponds to the central minimum point of the total potential $V_{\mathrm{Total}}^{+}\left(z\right)$. (**b**) The probability ${\left|\Psi_{0}^{-}\left(z\right)\right|}^{2}$ at the origin is zero, corresponding to the infinite total potential $V_{\mathrm{Total}}^{-}\left(z\right)$ at z=0.

The fact that the probability density function is inversely proportional to the total potential as introduced in (14) now can be visualized in Figure 4a,b which shows the ground-state probability density ${\left|\Psi_{0}^{\pm }\left(z\right)\right|}^{2}$ (black line) and total potential $V_{\mathrm{Total}}^{\pm }\left(z\right)$ (blue line). The total potential $V_{\mathrm{Total}}^{\pm }\left(z\right)$ have the form of

(51) $$V_{\mathrm{Total}}^{\pm }\left(z\right)=V\left(z\right)-\frac{d^{2}\ln\Psi_{0}^{\pm }\left(z\right)}{dz^{2}}$$

where $V\left(z\right)$ is given by (48) and the second term is the quantum potential $Q\left(z\right)$ corresponding to the ground-state wave function given by (5). Detailed information about the total potential can be obtained by finding the equilibrium point from the equation of motion (9),

(52) $$\frac{dz}{d\tau }=\frac{1}{i}\frac{d\ln\Psi_{0}^{\pm }\left(z\right)}{dz}$$

which yields equilibrium points $z_{\mathrm{eq}}^{+}=0,\;\;\pm 2.08331$ and $z_{\mathrm{eq}}^{-}=0,\;\;\pm 2.08367$. As shown in Figure 4, these equilibrium points are the positions where the two maxima of ${\left|\Psi_{0}^{\pm }\left(z\right)\right|}^{2}$ and the two minima of $V_{\mathrm{Total}}^{\pm }\left(z\right)$ locate, respectively. The largest difference between $V_{\mathrm{Total}}^{-}\left(z\right)$ and $V_{\mathrm{Total}}^{+}\left(z\right)$ is that at the origin z=0, where the total potential $V_{\mathrm{Total}}^{-}\left(z\right)$ approaches infinity, while the total potential $V_{\mathrm{Total}}^{+}\left(z\right)$ reaches its local minimum. In other words, it is impossible to observe the tunneling effect via the total potential $V_{\mathrm{Total}}^{-}\left(z\right)$ at z=0. When interpreted by probability, this means ${\left|\Psi_{0}^{-}\left(z\right)\right|}^{2}=0$ at z=0. In contrast, the probability ${\left|\Psi_{0}^{+}\left(z\right)\right|}^{2}$ evaluated at z=0 is ${\left|\Psi_{0}^{+}\left(0\right)\right|}^{2}=0.001938$, which is near zero but not equal to zero. Consequently, the potential $V_{\mathrm{Total}}^{+}\left(z\right)$ is not infinite at z=0, as shown in Figure 4a, allowing the tunneling motion to happen within $V_{\mathrm{Total}}^{+}\left(z\right)$.

Why is the nonzero probability at Rez=0 so important? The reason is that the position Rez=0 is where the equilateral triangle plane (symmetric plane) located. When the nitrogen atom passes through this symmetric plane and reaches the other side, the tunneling effect appears. For more than ten decades, physicists have regarded the tunneling effect as a typical quantum phenomenon which cannot be explained in a classical manner. The only way to realize this mysterious phenomenon relies on the probability interpretation provided by quantum mechanics. As the probability ${\left|\Psi_{0}^{+}\left(z\right)\right|}^{2}$ is not zero on the symmetric plane, it is possible for the nitrogen atom to pass repeatedly through the symmetric plane and reach the left apex and right apex to form the pyramid (ammonia) and the inverted pyramid (ammonia inversion) through the tunneling effect.

**Figure 5:**
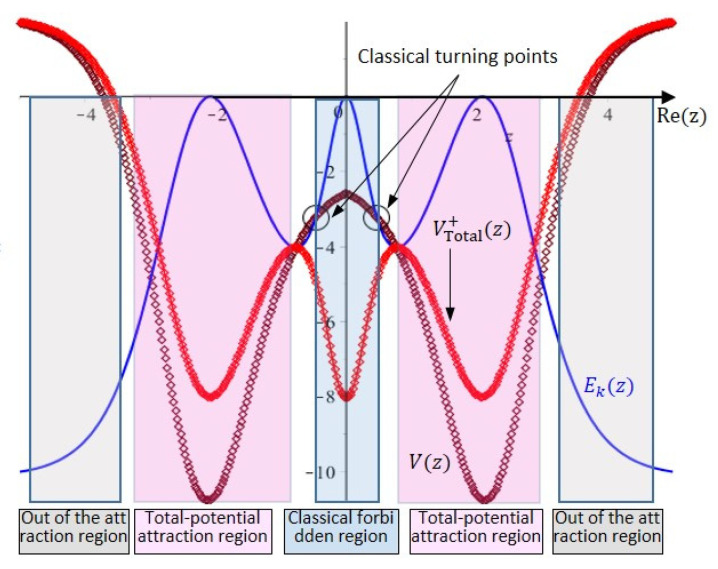
Three categories of regions in the total potential $V_{\mathrm{Total}}^{+}\left(z\right)$, the double-well potential $V\left(z\right)$, and the nitrogen atom’s kinetic energy $E_{k}\left(z\right).$ The classical forbidden region is defined by the classical turning points located at Rez=±0.468, where the double-well potential $V\left(z\right)$ is equal to the nitrogen atom’s kinetic energy $E_{k}\left(z\right).$ The two total-potential attraction regions are centered at the left and right minimum points of the total potential. Outside of the attraction regions, the attraction is too weak to pull the nitrogen atom back to the center of attraction.

Under the framework of quantum Hamilton mechanics, the wave function $\Psi_{0}^{+}\left(z\right)$ not only provides the tunneling probability, but also the tunneling trajectory and the potential barriers experienced by the nitrogen atom in the tunneling process. Figure 5 displays the spatial distributions of the total potential $V_{\mathrm{Total}}^{+}\left(z\right)$ (red line), the double-well potential $V\left(z\right)$ (brown line), and the nitrogen atom’s kinetic energy $E_{k}$ (blue line). As shown in Figure 5, the range of movement of the nitrogen atom can be divided into three regions: (1) the classical forbidden region, (2) the total-potential attraction regions, and (3) out of the total-potential attraction regions. The boundary of the classical forbidden region is determined by the classical turning points where the nitrogen atom’s kinetic energy $E_{k}$ equals the double-well potential $V\left(z\right)$. By letting $E_{k}=$ $V\left(z\right)$, the classical turning points are found to be Rez=±0.468.

**Figure 6:**
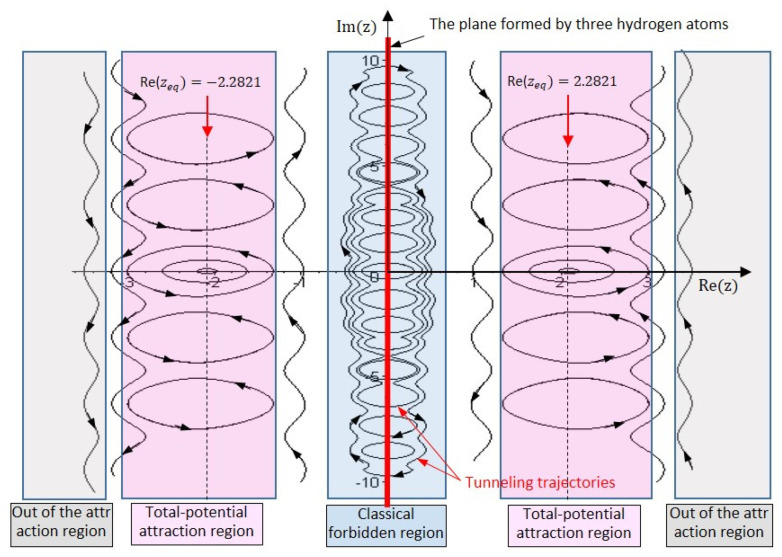
Complex trajectories in three types of regions of the nitrogen atom in the ground state. In the classical forbidden region, the closed trajectories are the tunneling trajectories crossing the symmetric plane from one side to the other side. In the total-potential attraction region, the trajectories enclose the equilibrium points at Rez=±2.2821, which are the minimum points of the total potential. The trajectories out of the attraction region are open trajectories.

The quantum trajectories in the three regions are determined by solving (52) with initial positions located in different regions, as shown in Figure 6. The trajectories in the classical forbidden region are closed paths encircling the points with Rez=0. If we inspect the quantum motion in this region along the real z−axis, we can see that the nitrogen atom oscillates across the symmetric plane formed by Rez=0 forming tunneling trajectories in the classical forbidden region. Other closed trajectories in Figure 6 appear on both sides of the symmetric plane and encircle their respective equilibrium points at Rez=±2.08331. This type of trajectory belongs to the total-potential attraction regions, where the motion of the nitrogen atom is under the attraction of the total potential $V_{\mathrm{Total}}^{+}\left(z\right)$ so that the center of the motion Rez=±2.08331 happens to be at the lowest point of the total potential $V_{\mathrm{Total}}^{+}\left(z\right)$. The trajectories out of the total-potential attraction regions are open since the attraction of the total potential in this region is too weak to maintain the rotation of the nitrogen atom around the equilibrium point.

**Figure 7:**
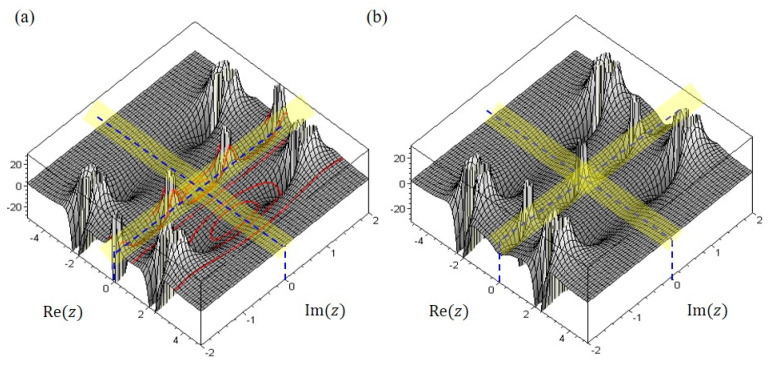
(**a**) The plot of the complex trajectories over the surface of the total potential $V_{\mathrm{Total}}^{+}\left(z\right)$, which has a local minimum at z=0 and allows the nitrogen atom to pass through. (**b**) The surface plot of the total potential $V_{\mathrm{Total}}^{-}\left(z\right)$, which goes to infinite at z=0 and forbids the nitrogen atom from passing through.

Figure 7 displays the complex trajectories (red curves) of the nitrogen atom over the surface of the total potential $V_{\mathrm{Total}}^{+}\left(z\right)$ and $V_{\mathrm{Total}}^{-}\left(z\right)$. The occurrence of tunneling through the barrier $V_{\mathrm{Total}}^{+}\left(z\right)$ at z=0 can be explained by the spatial distribution of the total potential $V_{\mathrm{Total}}^{+}\left(z\right)$, which has a local minimum at z=0, as Figure 6 and Figure 7a illustrate. On the other hand, Figure 7b shows that the total potential $V_{\mathrm{Total}}^{-}\left(z\right)$ is infinite around the origin z=0, which forbids the nitrogen atom from passing through from one side of the symmetric plane to the other, and so no tunneling effect can be observed. 

### 5.2. Tunneling Trajectory in the Excited States

Let us consider the first excited state in the same parameter setting. The parameters $a_{1}$ and $b_{1}$ are given by (25) and (26):

(53) $$a_{1}=-\beta /\left(\sqrt{4\gamma +1}-3\right)=-0.5913,\;\;b_{1}=\sqrt{\gamma +1/4}-3/2=1.8039$$

According to (29), the wave function $\Psi_{1}^{+}\left(z\right)$ of the first excited state is

(54) $$\Psi_{1}^{+}\left(z\right)=-e^{-0.5913\left(z-2.22\right)}{\left(\cosh\left(z-2.22\right)\right)}^{-1.8039}\left[0.2672+1.2672\tan h\left(z-2.22\right)\right]\;-e^{0.5913\left(z+2.22\right)}{\left(\cosh\left(z+2.22\right)\right)}^{-1.8039}\left[0.2672-1.2672\tan h\left(z+2.22\right)\right]$$

The accompanying wave functions $\Psi_{n}^{-}\left(z\right)$ all possess the property of $\Psi_{n}^{-}\left(0\right)=0$, which means there is zero probability of the tunneling motion occurring and thus it will not be considered in this section.

The equation of motion in the first excited state then is obtained by substituting $\Psi_{1}^{+}\left(z\right)$ into (9):

(55) $$\frac{dz}{d\tau }=\frac{1}{i}\frac{d\ln\Psi_{1}^{+}\left(z\right)}{dz}$$

**Figure 8:**
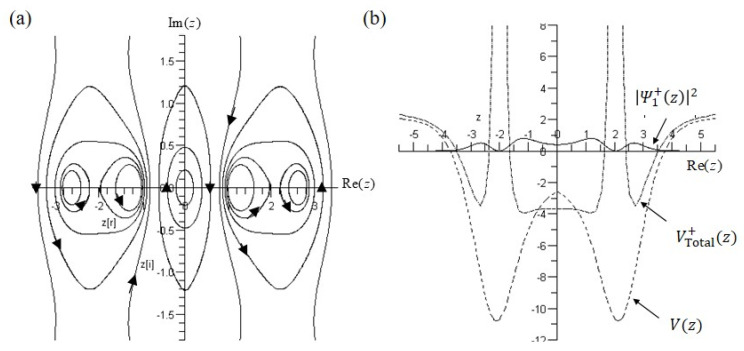
(**a**) The complex trajectories of the nitrogen atom in the first excited state with B=500 and C=2500. (**b**) The spatial distributions of the probability density ${\left|\Psi_{1}^{+}\left(z\right)\right|}^{2}$, the double-well potential $V\left(z\right)$, and the total potential $V_{\mathrm{Total}}^{+}\left(z\right)$.

There are five equilibrium points: $z_{\mathrm{eq}}^{+}=0$, ±1.179, and ±2.663, which coincide with the maxima and minima of the total potential

(56) $$V_{\mathrm{Total}}^{+}\left(z\right)=V\left(z\right)+Q\left(z\right)=V\left(z\right)-\frac{d^{2}\ln\Psi_{1}^{+}\left(z\right)}{dz^{2}}$$

with $V\left(z\right)$ given by (48). The quantum trajectories solved from (55) are shown in Figure 8a and the spatial distributions of $V_{\mathrm{Total}}^{+}\left(z\right)$ and $V\left(z\right)$ are shown in Figure 8b.

Compared with the ground-state tunneling trajectory, the nitrogen atom moves around Rez=0 with a much larger closed loop in the first excited state. This observation can be explained by the spatial distribution of the total potential $V_{\mathrm{Total}}^{+}\left(z\right)=V\left(z\right)+Q\left(z\right)$, which is quite flat around Rez=0. The total potential $V_{\mathrm{Total}}^{+}\left(z\right)$ has infinite hills at Rez=±2, which corresponds to the zero probability ${\left|\Psi_{1}^{+}\left(\pm 2\right)\right|}^{2}=0$, as predicted by the inverse proportionality (14). Without the participation of the quantum potential $Q\left(z\right)$, the double-well potential $V\left(z\right)$ alone cannot correctly explain the spatial distribution of ${\left|\Psi_{1}^{+}\left(z\right)\right|}^{2}$ as shown in Figure 8b.

Although the tunneling ability of the nitrogen atom is more significant in the first excited state than that in the ground state, the tunneling range does not envelop the equilibrium points on the both sides of the symmetric plane. This means that the complex trajectories shown in Figure 8a still do not exhibit the tunneling trajectory which oscillates between the two apexes of the pyramid. To search for such tunneling trajectory, we proceed to inspect the higher excited states. 

**Figure 9:**
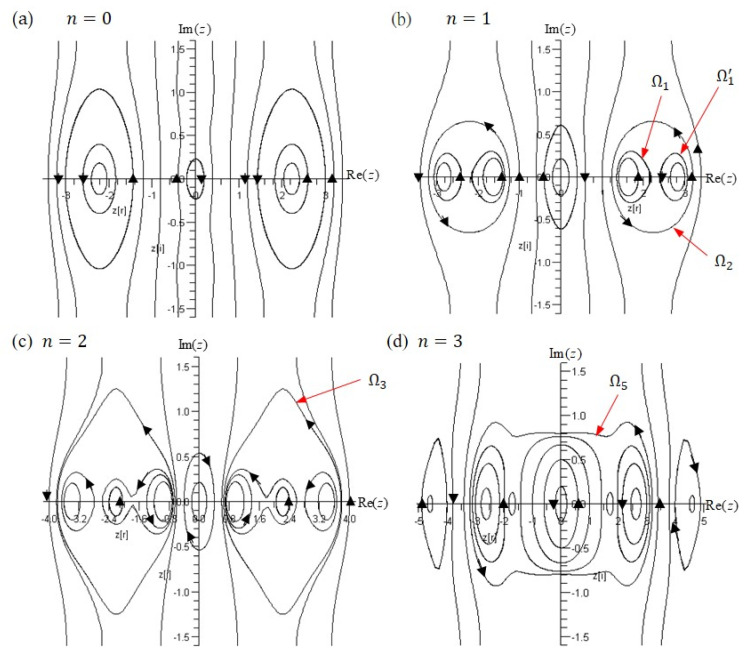
The complex trajectories of the nitrogen atom in the states n=0, 1, 2, 3 (**a**–**d**) with parameters B=5 and C=3000. The tunneling trajectory $\Omega_{5}$, which encloses both left and right equilibrium points, appears in the n=3 state.

The parameters we choose in the double-well potential, $B=500{\;\mathrm{cm}}^{-1}$ and $C=2500{\;\mathrm{cm}}^{-1}$, give us the number of bound states n≤1.77, according to (28). Thus, only the ground state (n=0) and the first excited state (n=1) are bounded in this potential. We have to change the values of B and C in order to find bound states with the quantum number n>1. Two new sets of B and C are examined and the results are tabulated in Table 1. Calculated from (28), the potential with $B=200{\;\mathrm{cm}}^{-1}$ and $C=2500{\;\mathrm{cm}}^{-1}$ has three bound states, while the potential with $B=5{\;\mathrm{cm}}^{-1}$ and $C=3000{\;\mathrm{cm}}^{-1}$ has four bound states. As listed in Table 1, every bound state has different equilibrium points, and exhibits different tunneling range and frequency. The trajectories in the four bound states corresponding to the case of $B=5{\;\mathrm{cm}}^{-1}$ and $C=3000{\;\mathrm{cm}}^{-1}$ are illustrated in Figure 9. It can be seen that for n≤2, the central tunneling trajectory across Rez=0 is separated from the right and left closed trajectories. A notable phenomenon appears in the n=3 state, where there are central tunneling trajectories covering five equilibrium points as shown in Figure 9d. This is the tunneling motion that oscillates between two apexes. However, the calculated period of oscillation is in the order of $10^{12}$ Hz, however the experimental value is of $10^{10}$ Hz. This discrepancy mainly arises from the neglect of the influence of the accompanying wave function $\Psi_{n}^{-}\left(z\right)$ on the tunneling process, and partially from the improper values of B and C used in the calculations, or from a modeling error incurred by using the double-well potential (15) to represent the potential experienced by the nitrogen atom in the ammonia molecule.

Oscillating trajectories form closed contours in the complex plane and their oscillation periods can be determined solely by the equilibrium points enclosed by the contours and are independent of their actual shape, according to the residue theorem [32]. Accordingly, oscillating trajectories enclosing different equilibrium points yields different oscillation periods. For example, we consider the three trajectory sets in Figure 9b in the n=1 state. The trajectory sets $\Omega_{1}$ and $\Omega_{1}^{’}$ comprise the trajectories enclosing one equilibrium point at $z_{\mathrm{eq}}=1.573$ and $z_{\mathrm{eq}}=2.861$, respectively. The trajectory set $\Omega_{2}$ comprises the trajectories enclosing two equilibrium points. Trajectories belonging to the same set have the same period of oscillation. The oscillation period of the trajectories in $\Omega_{2}$ is equal to the sum of the periods in $\Omega_{1}$ and $\Omega_{1}^{’}$. Obviously, a trajectory enclosing more equilibrium points has a larger range of oscillation and a longer period.

In Figure 9c, the trajectory in the set $\Omega_{3}$ encloses three equilibrium points and travels a large range between Rez=0.76 to 3.78. However, it does not pass through the symmetric plane Rez=0 from one side to the other. The tunneling trajectory, which encloses the equilibrium points on both sides of the symmetric plane, is found in the n=3 state. The trajectory set $\Omega_{5}$ in Figure 9d displays this kind of tunneling trajectory, which encircles five equilibrium points at $z_{\mathrm{eq}}=0,\pm 1.71$ and ±2.635. The nitrogen atom in this state repeatedly travels between Rez=−3.298 (x=−0.56Å) and Rez=3.145 (x=0.534Å) with the frequency $\nu =6.856\times 10^{12}\;\mathrm{Hz}$. 

There exist discrepancies of the tunneling range and tunneling frequency between our computation results and the experimental data, which are 0.38Å and $2.3786\times 10^{10}\;\mathrm{Hz}$, respectively. These discrepancies are as expected, because the tunneling motion we considered so far occurs in the stationary state $\Psi_{n}^{+}\left(z\right)$, but the actual tunneling motion in ammonia occurs in a transition state between $\Psi_{n}^{+}\left(z\right)$ and $\Psi_{n}^{-}\left(z\right)$. In the next section, we will consider tunneling motion in the transition state in order to reveal a trajectory description of ammonia’s tunneling dynamics that is closer to the experimental results.

## 6. Tunneling Dynamics in Two-Level Transition States

The oscillation of the nitrogen atom between two sides of the symmetric plane is called the two-level energy state transition, or tunneling in general. The ammonia inversion state has an energy level of $W_{0}^{-}=-1876.2959\;{\mathrm{cm}}^{-1}$ which is higher than that of the ammonia state $W_{0}^{+}=-1877.1078\;{\mathrm{cm}}^{-1}$ with an energy difference equal to $∆W_{0}=0.8119{\;\mathrm{cm}}^{-1}=1.0075\times 10^{-4}\;\mathrm{eV}$. To analyze the nitrogen atom in the two-level energy state, we consider the following time-dependent wave function [68],

(57) $$\Phi \left(z,\tau \right)=\frac{1}{\sqrt{2}}e^{iE_{0}\tau }\left[\left(\cos\left(\frac{A\tau }{2}\right)+\sin\left(\frac{A\tau }{2}\right)\right)\Psi_{0}^{+}\left(z\right)-\left(\cos\left(\frac{A\tau }{2}\right)-\sin\left(\frac{A\tau }{2}\right)\right)\Psi_{0}^{-}\left(z\right)\right]$$

where $\Psi_{0}^{+}\left(z\right)$ represents the ammonia state and $\Psi_{0}^{-}\left(z\right)$ represents the ammonia inversion state. The two parameters $E_{0}$ and A in (57) are defined as $E_{0}=\left(W_{0}^{-}+W_{0}^{+}\right)/2$ and $A=\left(W_{0}^{-}-W_{0}^{+}\right)/2$ so that the split energy levels in the ground state can be expressed as $W_{0}^{+}=E_{0}-A$ and $W_{0}^{-}=E_{0}+A$. According to (9), the equation of motion for the nitrogen atom in the transition state reads

(58) $$\frac{dz}{d\tau }=\frac{1}{i}\frac{d\ln\Phi \left(z,\tau \right)}{dz}$$

By solving (58), we can find the nitrogen atom’s tunneling trajectory. The initial value of the time-dependent wave function $\Phi \left(z,\tau \right)$ is

(59) $$\Phi \left(z,0\right)=\frac{1}{\sqrt{2}}\left(\Psi_{0}^{+}\left(z\right)-\Psi_{0}^{-}\left(z\right)\right)=\frac{1}{\sqrt{2}}\left(2\psi_{0}\left(-z-k\right)\right)$$

where we note $\Psi_{0}^{\pm }\left(z\right)=\frac{1}{\sqrt{2}}\left[\psi_{0}\left(z-k\right)\pm \psi_{0}\left(-z-k\right)\right]$ from (29) and (30). As $\Psi_{0}^{+}\left(z\right)$ has even symmetry, (59) can be expressed as

(60) $$\Phi \left(z,0\right)=\frac{1}{\sqrt{2}}\left[\psi_{0}\left(z-k\right)+\psi_{0}\left(-z-k\right)\right]=\Psi_{0}^{+}\left(z\right)$$

Since the initial state is the ammonia state $\Psi_{0}^{+}\left(z\right)$, the solution solved from (58) will show the tunneling process from the ammonia state $\Psi_{0}^{+}\left(z\right)$ to the ammonia inversion state $\Psi_{0}^{-}\left(z\right)$.

In the two-level transition state, the total potential $V_{\mathrm{Total}}\left(z,\tau \right)$ is not stationary but varies with time due to the time-dependent quantum potential $Q\left(z,\tau \right)$:

(61) $$V_{\mathrm{Total}}\left(z,\tau \right)=V_{s}\left(z\right)+Q\left(z,\tau \right)=V_{s}\left(z\right)-\frac{d^{2}\ln\Phi \left(z,\tau \right)}{dz^{2}}$$

**Figure 10:**
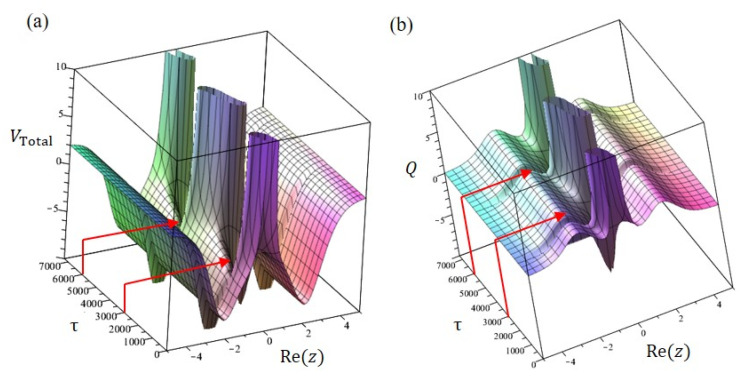
(**a**) The time evolution of the total potential $V_{\mathrm{Total}}$ shows that around τ=3000 and τ=6000 the gap created by the reduction of the potential barrier (marked by the red arrows) forms a tunneling channel, allowing the nitrogen atom to pass through the potential barrier to the other side. (**b**) The change of the quantum potential Q over time controls the formation of tunneling channels.

Figure 10a,b illustrates how the total potential and quantum potential alter their shapes with time. As time proceeds, we can observe that the potential hill at Rez=0 becomes the well around τ=3000. The nitrogen atom can pass through the region where the potential hill becomes the well. In other words, the quantum potential produces some channels in a specific time, allowing the nitrogen atom to transit from one side of the symmetric plane to the other side as shown in Figure 10b.

**Figure 11:**
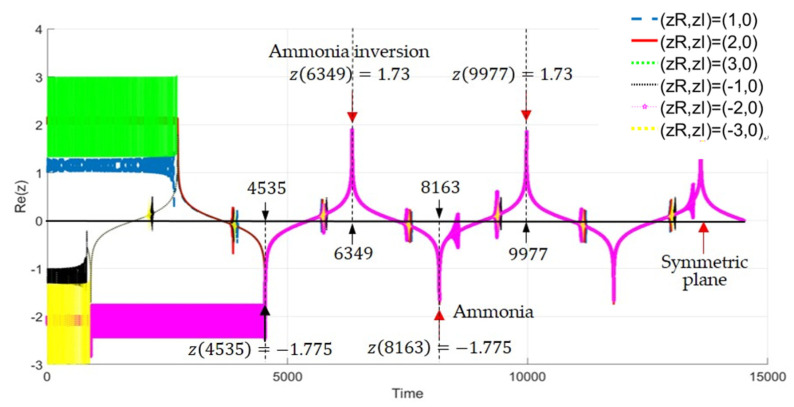
The tunneling trajectories launched from different initial positions. The apex of the ammonia state and the ammonia inversion state are, respectively, at Rez=−1.775 and Rez=1.73. The time interval between each apex transition is ∆τ=1814, corresponding to the actual time interval $∆t=2.0529\times 10^{-11}$ s. A complete tunneling period is ∆τ=3628, and the tunneling frequency is 24.35 GHz.

Figure 11 displays the time response of the nitrogen atom’s position Rez. The figure shows six trajectories starting from different initial positions, which are represented by six different colors. These different trajectories eventually coincide together, showing the same amplitude and the same period of oscillation. It can be observed that the nitrogen atom oscillates between Rez=−1.775 $\left(0.30175Å\right)$ and Rez=1.73 $\left(0.294Å\right)$ with a fixed period of oscillation. According to the computation data, the time interval between each apex transition is ∆τ=1814 and a complete period is $\bar{T}=2∆\tau =3628$. When converted to an actual time unit, the tunneling period is found to be $4.106\times 10^{-11}$ s and the corresponding tunneling frequency is 24.35 GHz, which is close to the measured frequency 23.8 GHz, as compared in Table 2. The theoretical frequency listed in Table 2 is calculated from the formula $∆W_{0}=W_{0}^{-}-W_{0}^{+}=h\nu$, which yields ν=24.35 GHz, the same as that computed from the tunneling dynamics.

The measured tunneling range is 0.38 Å, however our computation shows that the tunneling ranges on both sides of the symmetrical plane are not equal. As shown in Figure 11, the tunneling range in the ammonia state is Rez=−1.775 x=−0.30175 Å, while that in the ammonia inversion state is Rez=1.73 x=0.294 Å. This result is consistent with the fact that the energy level of the ammonia inversion state is slightly higher than that of the ammonia state. This energy-level difference causes the total potential $V_{\mathrm{Total}}\left(z\right)$ on the right side of the symmetric plane to be higher than that of the left-hand side. Therefore, the nitrogen atom encounters a higher potential barrier on the right side of the total potential so that its tunneling length is reduced slightly.

Observing the six trajectories in Figure 11, we can see that the black trajectory enters the tunneling process first, and the pink trajectory last. In other words, when the nitrogen atom starts from a different initial position, the time at which it can cross the symmetry plane of the ammonia molecule is also different, that is, the time at which the tunneling mechanism is triggered is different. The trigger point of the tunneling mechanism is determined by the time when the tunneling channel appears in the total potential. It can be seen from Figure 10 that the time when the tunneling channel first appeared in the total potential is about τ=3000. Therefore, the nitrogen atom must wait on one side of the symmetric plane until the tunneling channel appears at time τ=3000 before passing through the plane to the other side. However, the shape of the total potential in Figure 10 is only applicable to the nitrogen atom starting from a certain point. If starting from a different point, the nitrogen atom will face different shapes of total potential, thus the time at which the tunneling channel will appear also changes.

**Figure 12:**
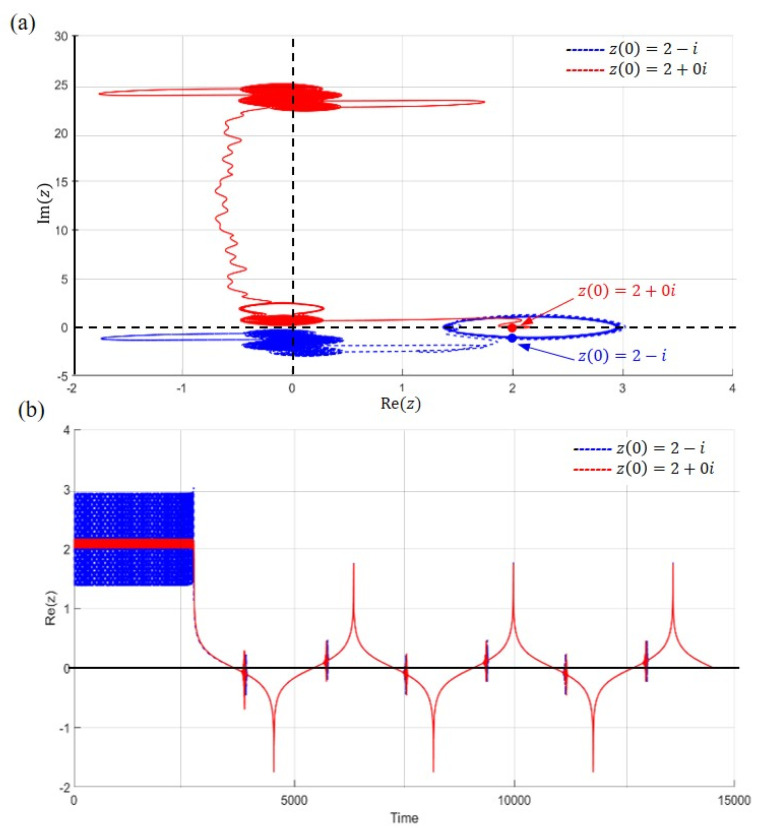
The tunneling motion in the complex plane with initial positions $z\left(0\right)=2+0i$ (solid red line) and $z\left(0\right)=2-i$ (dash blue line). (**a**) The trajectory with the initial position $z\left(0\right)=2+0i$ starts to tunnel around Imz=24, while the trajectory with the initial position $z\left(0\right)=2-i$ has a tunneling trajectory that is more close to the real z−axis. (**b**) As viewed along the real axis, both trajectories start the tunneling process at the same time and have the same tunneling period.

The actual tunneling motion occurs in the complex plane, however the physical measurement can only achieve its projection on the real axis. Figure 12 shows two tunneling trajectories in the complex plane, which are obtained by solving (58) with two initial positions $z\left(0\right)=2+0i$ and $z\left(0\right)=2-i$. It is noteworthy that the nitrogen atoms have different trajectories even there real part of the initial positions are the same. An interesting phenomenon occurs when the initial position is closer to the real axis, as the area where the tunneling trajectory occurs will be farther away from the real axis, as illustrated in Figure 12a. Although tunneling trajectories in the complex plane undergo various irregular changes due to their uncertain initial positions, once they are projected onto the real axis, they all overlap onto the same trajectory (as shown in Figure 11 and Figure 12b). This result explains why our measurements of ammonia molecules always achieve consistency.

**Figure 13:**
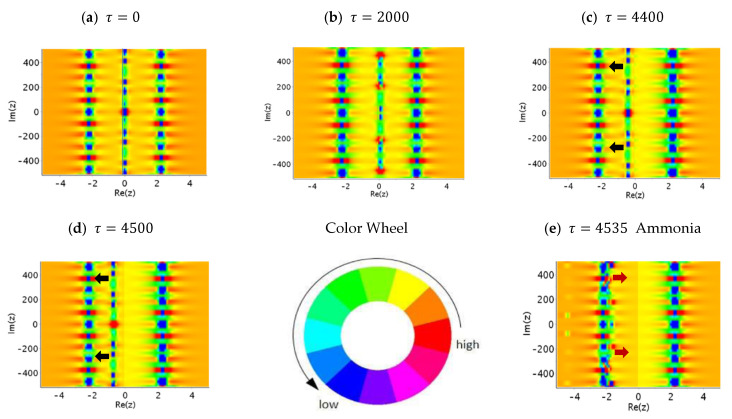

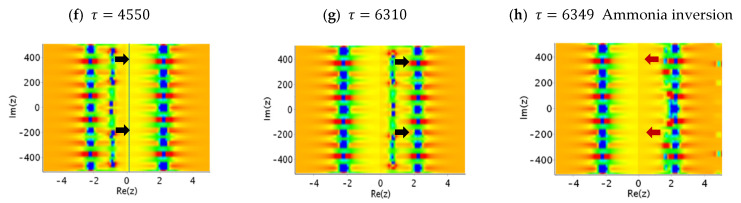
Snapshots of the total potential over the complex plane at several moments. The height of the total potential is labelled by different colors as indicated by the color wheel. The central tunneling channel is stationary in the time interval 0≤τ≤4000 (as (**a**,**b**) show). After τ=4000, the channel starts to move to the left-hand side (the black arrows in (**c**,**d**) show the moving direction), and reaches the pyramid apex (the ammonia state) at τ=4535 as (**e**) shows. Then the central tunneling channel changes direction (see the red arrows in (**e**)) and moves back to the right-hand side of the symmetric plane (refers to (**f**,**g**)), meeting the inverted pyramid apex (the ammonia inversion state) at τ=6349 as shown in (**h**). Then it immediately changes direction (see the red arrows in (**h**)) and moves back to the left-hand side. (**e**–**h**) illustrate a half cycle of the ammonia and ammonia inversion transition. After τ=6349, the tunneling channel moves to the left-hand side in the same process as shown in (**f**,**g**). The tunneling channel varies periodically between the two states with associated tunneling trajectory and arrival time, as shown in Figure 11. During the tunneling process, the nitrogen atom rides on the tunneling channel and moves along with it.

From the above analysis, we realize that the tunneling motion of the nitrogen atom in ammonia is carried out through the tunneling channel of the total potential. In order to know the changes in the tunneling channel during the entire tunneling process, we illustrate the distribution of the total potential on the complex plane at several different moments in a series of subgraphs in Figure 13. In this figure, the height of the total potential is indicated by color so that the potential from high to low is sequentially marked by red, green, and blue, as labelled by the attached color wheel. In each subgraph, there are three vertical lines connected by many green and blue spots. The position of these three lines represents the place where the total potential is lower, and it is also the place where the nitrogen atom is more likely to appear. The vertical lines on the left and right sides are fixed and represent the equilibrium positions of the nitrogen atom on the two sides of the symmetry plane. The vertical line in the middle moves with time, which is what we call the tunneling channel. The blue spots on the tunneling channel are the places where the total potential is the lowest. The nitrogen atom rides on these blue spots and moves along with the tunneling channel.

As can be seen from Figure 13, the central tunneling channel is stationary in the time interval 0≤τ≤4000. This is the waiting time required to trigger the tunneling mechanism, as mentioned in Figure 11. After τ=4000, the channel starts to move to the left-hand side, and reach the left equilibrium point Rezeq=−1.775 at τ=4535. After that, the central tunneling channel moves back to the right-hand side of the symmetric plane and meets the right equilibrium point Rez=1.73 in the ammonia inversion state at τ=6349. Accordingly, the central tunneling channel moves periodically between the two sides of the equilibrium points, which means that the nitrogen atom oscillates between the two sides of the apexes, tunneling from ammonia to ammonia inversion.

## 7. Conclusions

Quantum Hamilton mechanics provides a remarkable deterministic way to explore tunneling dynamics in ammonia. The magic phenomenon that a nitrogen atom with insufficient kinetic energy can pass through the potential barrier in ammonia now becomes understandable by tracing the trajectory of the nitrogen atom. We found that the quantum potential plays the most important role in tunneling dynamics by creating a tunneling channel in the complex plane for the nitrogen atom to pass through. The tunneling phenomenon as traditionally described in the language of probability can now be described in more detail through the motion of the nitrogen atom in a complex plane.

Complex tunneling trajectories in both the stationary states and the two-level transition state in the double-well potential have been studied here. Tunneling dynamics in stationary states have an analytical expression and help us to describe the tunneling phenomenon concisely. However, the computed tunneling frequency and tunneling range in the stationary states have a significant deviation from the experimental data. Compared to the stationary states, the two-level transition state provides a more accurate description of the tunneling process, if the double-well potential is used. In the two-level state, we find that a tunneling channel that moves with time in the complex plane appears in the total potential. It is through this moving channel that the nitrogen atom travels back and forth between the ammonia state and the ammonia inversion state. The tunneling frequency, 24.35 GHz, computed from the complex trajectory of the nitrogen atom, is close to the measured frequency of 23.8 GHz. 

As to the tunneling range, the measured value is 0.38 Å, however our analysis shows that the tunneling ranges on the two states should be different. The reason is that the potential barrier in the ammonia inversion state is slightly higher than that in the ammonia state and thus it is harder for the nitrogen atom to penetrate. Accordingly, the tunneling range in the ammonia inversion state is found to be 0.294 Å, which is slightly shorter than that in the ammonia state, which is found to be 0.30175 Å. This asymmetric apex arises from the experimental observed energy split of the ammonia state and its inversion state. In this study, we analyze the transition between the two states in detail and propose a theoretical result of the different tunneling ranges in the two states. Hopefully, this study can provide some useful information for related experiments.

## Figures and Tables

**Table 1:** The computation results of the ammonia vibration in several eigenstates.

		B=500, C=2500	B=500, C=2500	B=5, C=3000
n=0	Equilibrium Positions	0,±2.0830	0, ±2.166	0, ±2.219
	Tunneling Range	−0.468~0.468	−0.334~0.334	−0.287~0.287
	Tunneling Frequency	$7.638\times 10^{13}$ Hz	$9.224\times 10^{13}$ Hz	$1.277\times 10^{14}$ Hz
n=1	Equilibrium Positions	0,±1.179,±2.663	0,±1.406,±2.806	0,±1.573,±2.861
	Tunneling Range	−0.602~0.602	−0.530~0.530-	−0.416~0.4160
	Tunneling Frequency	$1.446\times 10^{13}$ Hz	$2.792\times 10^{13}$ Hz	$5.415\times 10^{13}$ Hz
n=2	Equilibrium Positions	-	0,±2.020,±3.167	0,±0.870,±2.223,±3.440
	Tunneling Range	-	−1.956~1.956	−0.532~0.532
	Tunneling Frequency	-	$5.584\times 10^{12}$ Hz	$1.629\times 10^{13}$ Hz
n=3	Equilibrium Positions	-	-	0,±1.710,±2.635,±4.602
	Tunneling Range	-	-	−3.298~−3.145, 3.145~3.298
				−1.509~1.509
	Tunneling Frequency	-	-	$6.856\times 10^{12}$ Hz
				$8.285\times 10^{12}$ Hz

**Table 2:** Comparisons of the experimental data with the theoretical and computation results of the two-level energy state.

Comparison	Tunneling Range	Energy Splitting	Tunneling Frequency
Experimental data	0.38 Å	$9.8\times 10^{-5}\;\mathrm{eV}$	23.8 GHz
Theoretical results	-	$10.0725\times 10^{-5}\;\mathrm{eV}$	24.35 GHz
Computation results	0.3017 Å	$10.0725\times 10^{-5}\;\mathrm{eV}$	24.35 GHz

## Data Availability

Not applicable.
